# Associations of unhealthy lifestyles with metabolic syndrome in Chinese rural aged females

**DOI:** 10.1038/s41598-020-59607-x

**Published:** 2020-02-17

**Authors:** Yuming Wang, Runqi Tu, Huijuan Yuan, Lijun Shen, Jian Hou, Xiaotian Liu, Miaomiao Niu, Zhihan Zhai, Mingming Pan, Chongjian Wang

**Affiliations:** 1 Henan Provincial People’s Hospital, Zhengzhou, Henan P.R. China; 2 Zhengzhou University People’s Hospital, Zhengzhou, Henan P.R. China; 3 Department of Epidemiology and Biostatistics, College of Public Health, Zhengzhou University, Zhengzhou, Henan P.R. China

**Keywords:** Metabolic syndrome, Risk factors

## Abstract

The purpose of this study is to update the prevalence of metabolic syndrome (MetS) and explore to identify the susceptible populations. A total of 38208 subjects aged 18 to 79 years were obtained from the Henan Rural Cohort Study (n = 39259). Five criteria (ATP β, IDF, JIS, CDS, EGIR) were used to estimate the prevalence of MetS. Multivariate logistic regression analysis was used to assess odds ratios (ORs) and 95% confidence interval (CI) of potential risk factors with MetS. The age-standardized prevalence of MetS were 27.87%, 24.63%, 27.40%, 18.00% and 8.91% according to the standard of ATP β, IDF, JIS, CDS, and EGIR, respectively. After adjusted for the potential confounding factors, aging, females, physical activity and the state of drinking were independent risk factors of MetS. MetS is positively associated with stroke and coronary heart disease in all five criteria (*P* < 0.01). The current data identify a high prevalence of MetS among Chinese rural adults. Especially for aged females with unhealthy lifestyle had a higher risk for MetS.

## Introduction

Metabolic syndrome (MetS) is a multifaceted disorders, which includes abdominal obesity, high blood pressure, dyslipidemia, and hyperglycemia, and has been coded in the International Classification of Diseases^1^. Both insulin resistance and obesity are major risk factors for MetS^2,3^. Based on this, MetS have been defined by different scientific organizations such as National Cholesterol Education Program Adult Treatment Panel β (ATP β)^4^, International Diabetes Federation (IDF)^5^, a Joint Interim Statement of the International Diabetes Federation Task Force on Epidemiology and Prevention; National Heart, Lung, and Blood Institute; American Heart Association; World Heart Federation; International Atherosclerosis Society; and International Association for the Study of Obesity (JIS)^6^, Chinese Diabetes Society (CDS)^7^, European Group for the Study of Insulin Resistance (EGIR)^8^. Thus, the prevalence of MetS were evaluated using the mentioned criterions of MetS in different international organizations.

The prevalence of the MetS had been reported among Chinese population, according to the various diagnostic criterions. For instance, the prevalence of MetS was 21.3% (ATP β), 18.2% (IDF), 10.5% (CDS) in the Chinese population from nine provinces in 2009^9^, and in urban area of northeast China, the overall prevalence of MetS is 27.4%, with 27.9% in men and 26.8% in women^10^. Another study showed that in 2009, the prevalence of MetS among adults was 21.33% (IDF) in rural areas of Xinjiang province^11^. From those researches, the results remain inconsistent. Briefly, the mainly reason was the population differences: one of the studies population was from multi-ethnic adults in rural areas in Xinjiang provinces of China^11^; another study population was from urban areas of northeast China^10^; the last study population was from nine provinces of China (Liaoning, Heilongjiang, Jiangsu, Shandong, Henan, Hubei, Hunan, Guangxi and Guizhou)^9^. However, the epidemic characteristics of MetS in rural areas were remained to be explored. Due to that the economic and urbanization development these years, the lifestyles had been greatly changed in the rural areas, which may contribute to increase the risk of MetS. A recent study indicated that individuals with MetS had a 2-fold or 5-fold increase the risk for CVD or type 2 diabetes, compared to individuals without MetS^12^. Moreover, evidence showed that MetS was related to the increased all-cause mortality^13^. It was urgently to update the prevalence of MetS and aid to make suitable policy for administration the MetS. Therefore, the objective of current study were: (1) to update the prevalence and the risk factors of MetS, (2) to explore the epidemic characteristics of susceptible populations with MetS.

## Participants and Method

### Settings and participants

This study was conducted from 2015 to 2017 in 5 rural regions (suiping country, Yuzhou country, Xinxiang country, Tongxu country, and Yima country) of Henan province^14^. The target population were permanent residents aged 18–79 years. Those people whose information about the biochemical indicators were deficient were excluded. Ultimately, 38208 participants completed questionnaires, anthropometric measurements and blood tests.

### Data collection

Each individual completed a standard questionnaire included information on general demographic characteristics, lifestyles, histories of diseases by a face-to-face interview. The education levels were classified into illiteracy, primary school, junior middle school, senior middle school or above groups. The marital status were categorized into married/cohabitation, unmarried/divorced/widowed. The smoking status were classified into current smoker (a person who smoked more than one cigarette per day in the past 6 months) and never smoker. Current drinker was defined as a person who intake alcoholic beverages (spirits, beer, wine, and other alcohol beverages) at least 12 times in the past one year. According to Chinese dietary guidelines^15^, more than 500 g vegetable and fruit daily consumption was defined as more vegetable and fruit intake. A person was considered as high fat diet, who ate more than 75 g meat daily. From the international physical activity questionnaire (IPAQ)^16,17^, physical activity was categorized into three levels: low, moderate, and high. The waist circumference was measured at 1 cm above the navel in a horizontal plane by a non-elastic tape to the nearest 0.1 cm. Height of participants was measured twice without shoes using a wall-mounted ruler tape with an insertion buckle at one end to the nearest 0.1 cm. Body weight was measured once with the subjects in light clothing and barefoot using mechanical scale on a level surface to the nearest 0.1 kg^18^. Body mass index (BMI) was calculated as weight in kilograms divided by the square of height in meters. Blood pressure was measured by using electronic sphygmomanometer (Omron HEM-7071A, Japan). All participants were required to have a seat at least 5 minutes and then blood pressure were measured for three times and the averaged of three measurement values was used for analysis.

### Definitions

Four worldwide definitions and one Chinese criterion were used to diagnose MetS. These definitions were from the ATP β^4^, IDF^5^, JIS^6^, CDS^7^, and EGIR^8^. These criteria were presented in Supplementary Table 1.

### Statement

All methods were carried out based on relevant guidelines and regulations. Ethics approval was obtained from the “Zhengzhou University Life Science Ethics Committee”, and written informed consent was obtained for all participants before conducted this study. Ethic approval code: [2015] MEC (S128). and all participants provided written informed consent.

### Statistical analysis

Continuous variables were presented as mean ± standard deviation (SD). Differences between groups were tested using t-test. Categorical variables were expressed as numbers and percentages and analyzed by using chi-square test. Based on Chinese population data from 2010 census, direct standardization was used to calculate the age-standardized prevalence of MetS. The multivariate logistic regression models were used to calculate ORs and 95% CI of associations between the potential factors and MetS. Statistical analysis was considered statistically significant when the *P* value of two-tailed was set at less than 0.05. The data were analyzed using SAS9.1 software package (SAS Institute, USA) and R version 3.5.1.

## Result

### Demographic characteristics

The demographic characteristics of the 38208 participants (male 14877, female 23331) aged 18–79 years old were showed in Table 1. The mean (SD) age of all participants was 55.57 (12.18) years old. The males are more likely to have younger age, eat high-fat diet, have a higher proportion of current smokers or current drinkers as well as a higher level of height, waist circumference (WC), weight, systolic blood pressure (SBP) and diastolic blood pressure (DBP). While females are more likely to eat more vegetable and fruit intake, have a higher level of body mass index (BMI), high density lipoprotein cholesterol (HDL-C), low density lipoprotein cholesterol (LDL-C), total cholesterol (TC) and insulin (INS) (*P* < 0.001). The differences in distributions of the culture, income and physical activity were found between males and females (all *P* < 0.001). No difference in the variables of marriage status, and the level of triglyceride (TG), glucose (GLU) were found between males and females (all *P* > 0.05).

**Table 1:** Characteristics of the participants.

Variables	Total (38208)	Male (14877)	Female (23331)	P^*^
Age (years), mean ± SD	55.57 ± 12.18	56.59 ± 12.31	54.92 ± 12.05	<0.001
Culture, n (%)				<0.001
Illiteracy	6388 (16.72)	1277 (8.58)	5111 (21.91)	
Primary school	10775 (28.20)	3760 (25.27)	7015 (30.07)	
Junior middle school	15207 (39.80)	6877 (46.23)	8330 (35.70)	
≥Senior middle school	5838 (15.28)	2963 (19.92)	2875 (12.32)	
Marriage status, n (%)				0.460
Married/cohabitation	34306 (89.79)	13379 (89.93)	20927 (89.70)	
Divorced/widowed/unmarried	3902 (10.21)	1498 (10.07)	2404 (10.30)	
Income, n (%)				<0.001
<500 RMB	13593 (35.58)	5360 (36.03)	8233 (35.29)	
500–999 RMB	12578 (32.92)	4727 (31.77)	7851 (33.65)	
1000–1999 RMB	9197 (24.07)	3552 (23.88)	5645 (24.20)	
≥2000 RMB	2840 (7.43)	1238 (8.32)	1602 (6.87)	
Physical activity, n (%)				<0.001
Low	12291 (32.17)	5292 (35.57)	6999 (30.00)	
Moderate	14537 (38.05)	4166 (28.00)	10371 (44.45)	
High	11380 (29.78)	5419 (36.43)	596 1(25.55)	
More vegetable and fruit intake, n(%)	16013 (41.91)	6406 (43.06)	9607 (41.18)	<0.001
High-fat diet, n(%)	7336 (19.20)	3766 (25.31)	3570 (15.30)	<0.001
Current smoker, n (%)	7145 (18.70)	7080 (47.59)	65 (0.28)	<0.001
Current drinker, n (%)	6778 (17.74)	6167 (41.45)	611 (2.62)	<0.001
Height(cm), mean ± SD	159.65 ± 8.18	166.58 ± 6.30	155.24 ± 5.85	<0.001
WC(cm), mean ± SD	84.11 ± 10.37	85.67 ± 10.56	83.12 ± 10.13	<0.001
Weight(kg), mean ± SD	63.51 ± 11.09	68.41 ± 11.41	60.39 ± 9.66	<0.001
BMI(kg/m^2^), mean ± SD	24.86 ± 3.55	24.59 ± 3.47	25.03 ± 3.59	<0.001
SBP(mmHg), mean ± SD	125.90 ± 20.00	126.61 ± 18.59	125.45 ± 20.84	<0.001
DBP(mmHg), mean ± SD	77.67 ± 11.63	78.75 ± 11.92	76.98 ± 11.39	<0.001
HDL-C(mmol/L), mean ± SD	1.33 ± 0.33	1.26 ± 0.32	1.37 ± 0.33	<0.001
LDL-C(mmol/L), mean ± SD	2.88 ± 0.82	2.83 ± 0.80	2.90 ± 0.83	<0.001
TG(mmol/L), mean ± SD	1.68 ± 1.13	1.67 ± 1.16	1.68 ± 1.10	0.196
TC(mmol/L), mean ± SD	4.75 ± 0.97	4.63 ± 0.94	4.83 ± 0.99	<0.001
INS(mIU/mL), mean ± SD	10.89 ± 5.32	10.66 ± 5.36	11.03 ± 5.28	<0.001
GLU(mmpl/mL), mean ± SD	5.53 ± 1.48	5.52 ± 1.47	5.54 ± 1.49	0.135

Abbreviations: SD, standard deviation; WC, waist circumference; BMI, body mass index; SBP, systolic blood pressure; DBP, diastolic blood pressure; HDL-C, high density lipoprotein cholesterol; LDL-C, low density lipoprotein cholesterol; TG, triglyceride; TC, total cholesterol; INS, insulin; GLU, glucose.

*Compared between male and female.

### The prevalence of MetS and its components

**Figure 1:**
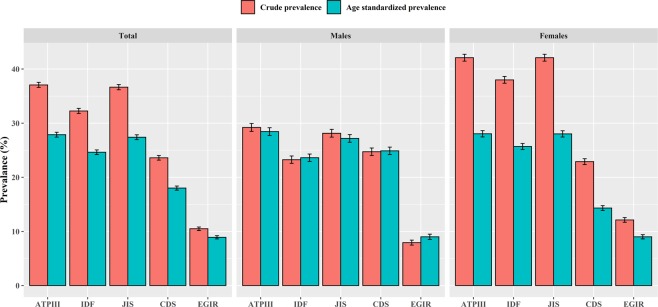
Crude and age Standardized prevalence of MetS based on five diagnostic criteria in Henan rural adults.

**Figure 2:**
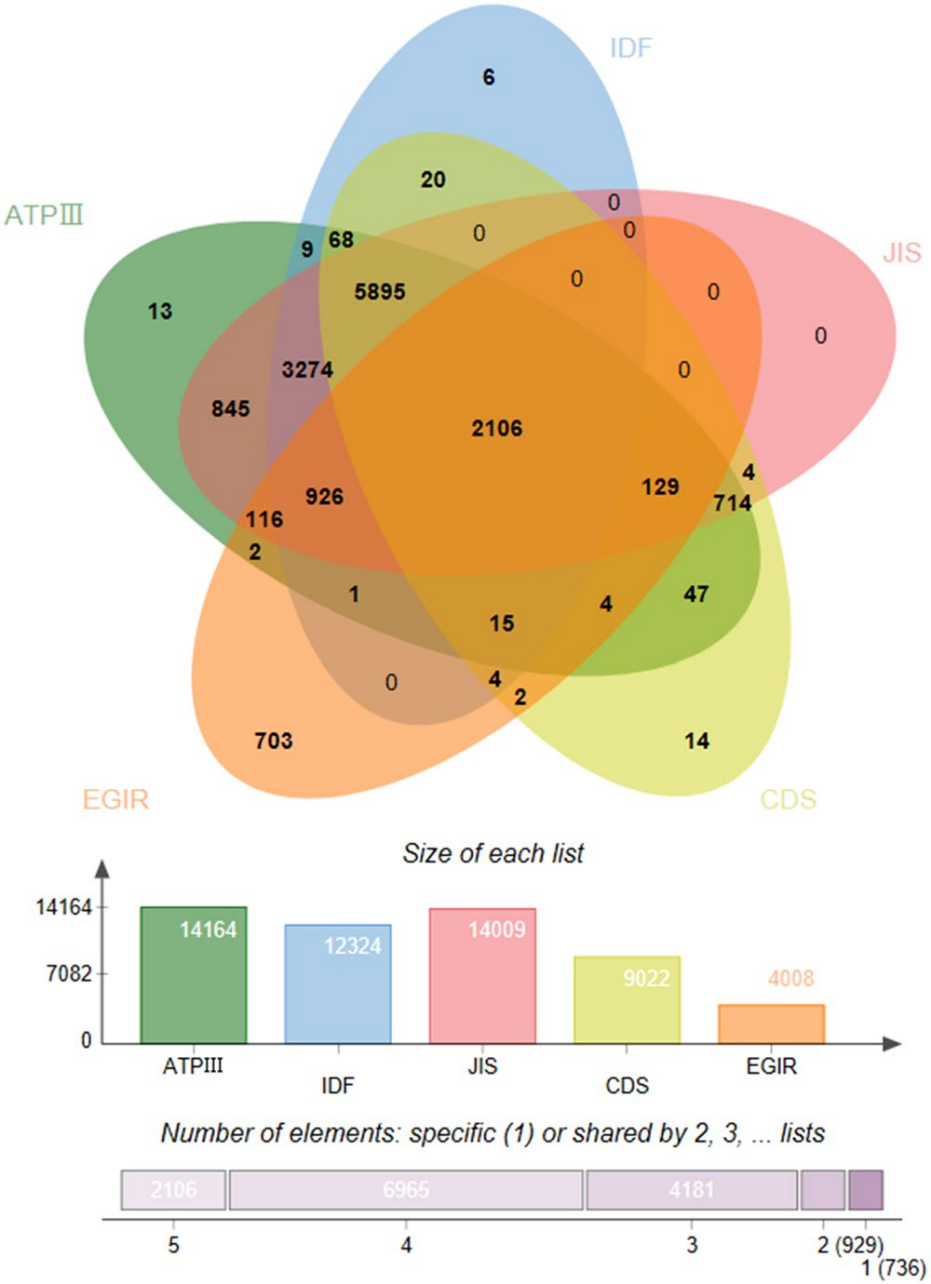
The overlap of MetS defined by different diagnostic criteria.

The crude prevalent MetS of the criteria of ATP β, IDF, JIS, CDS, and EGIR were 37.07%, 32.26%, 36.65%, 23.60%, and 10.49%, respectively. The age-standardized prevalent MetS of the criteria of ATP β, IDF, JIS, CDS, and EGIR were 27.87%, 24.63%, 27.40%, 18.00%, and 8.91%, respectively. The prevalence of MetS in females was higher than males for the other criteria except for the CDS **(**Fig. 1**)**. The overlap of MetS defined by the five diagnostic criteria was shown in Fig. 2. Only 2106 participants were identified as MetS satisfied with all five criteria. Based on this, comparison of the authenticity of each criteria referring the CDS criterion **(**Supplementary Table 2**)**. The ATP β, IDF, JIS had a higher than EGIR about sensitivity and Youden’s index. While for the specificity, The ATP β, IDF, JIS had a lower level than EGIR.

**Figure 3:**
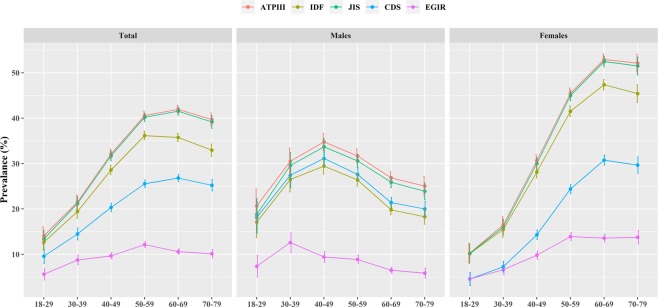
Age-specific prevalence of MetS based on five diagnostic criteria in Henan rural adults.

**Figure 4:**
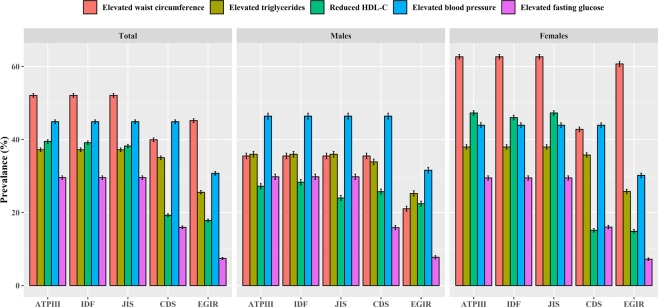
The prevalence of the components of MetS based on five diagnostic criteria in Henan rural adults.

The result about the components showed that all participants aged 60–69 years, males aged 40–49 years, females aged 70–79 years, had the highest prevalence of MetS in the other criteria except for the EGIR, rather than individuals with other aged groups **(**Fig. 3). Among five components of MetS, the highest prevalence of increased waist circumference were observed among all participants and females as well as the highest prevalence of elevated blood pressure were exhibited among males in the other criteria except for the CDS **(**Fig. 4).

### Influence factors of MetS

Table 2 exhibited the odds ratios of associations of potential influencing factors with MetS. Female, old age, current alcohol intake, lack of physical activity and non-cigarette smoking were significantly positively associated with the prevalence of MetS. For the marriage status, the divorced, widowed, and unmarried were a protective factor for MetS in the other four criteria, except for EGIR. Associations of higher vegetables intake with MetS were not incompatible based on the criteria of ATP β, IDF, JIS, CDS, and EGIR.

**Table 2:** Association between potential risk factors and MetS based on different criteria among Henan rural adults.

Variables	OR (95% *CI*)*				
	ATP β	IDF	JIS	CDS	EGIR
**Age (year)**					
18-	1.00	1.00	1.00	1.00	1.00
30-	1.74 (1.45,2.09)	1.72 (1.42,2.08)	1.82 (1.51,2.20)	1.67 (1.34,2.07)	1.62 (1.23,2.14)
40-	3.14 (2.64,3.72)	3.00 (2.51,3.58)	3.27 (2.74,3.88)	2.56 (2.10,3.13)	1.88 (1.45,2.43)
50-	4.68 (3.97,5.53)	4.42 (3.71,5.26)	4.90 (4.13,5.80)	3.56 (2.93,4.33)	2.61 (2.03,3.35)
60-	5.07 (4.28,6.00)	4.52 (3.79,5.40)	5.31 (4.47,6.32)	3.66 (3.01,4.46)	2.30 (1.78,2.97)
70–79	4.47 (3.74,5.34)	3.93 (3.26,4.73)	4.68 (3.90,5.60)	3.17 (2.58,3.89)	2.15(1.64,2.81)
Female	1.81 (1.70,1.92)	2.17 (2.04,2.32)	1.91 (1.80,2.03)	0.93 (0.87,0.99)	1.51 (1.36,1.66)
**Education level**					
Illiteracy	1.00	1.00	1.00	1.00	1.00
Primary school	0.99 (0.93,1.06)	1.00 (0.94,1.07)	0.98 (0.92,1.05)	1.01 (0.94,1.09)	0.92 (0.83,1.02)
Junior middle school	0.94 (0.88,1.00)	0.95 (0.88,1.02)	0.94 (0.88,1.01)	0.90 (0.84,0.98)	0.78 (0.70,0.87)
≥Senior middle school	1.03 (0.94,1.12)	1.04 (0.95,1.14)	1.02 (0.94,1.12)	1.00 (0.91,1.10)	0.81 (0.71,0.92)
**Marital status**					
Married/cohabiting	1.00	1.00	1.00	1.00	1.00
Divorced/widowed/unmarried	0.91 (0.84,0.98)	0.88 (0.82,0.95)	0.90 (0.84,0.97)	0.87 (0.80,0.95)	0.93 (0.83,1.04)
**Per capita monthly income**					
<500 RMB	1.00	1.00	1.00	1.00	1.00
500–999 RMB	1.08 (1.03,1.14)	1.10 (1.04,1.16)	1.09 (1.03,1.14)	1.06 (1.00,1.13)	0.97 (0.90,1.06)
1000–1999 RMB	1.08 (1.02,1.15)	1.08 (1.02,1.15)	1.08 (1.02,1.15)	1.05 (0.99,1.12)	1.09 (1.00,1.19)
≥2000 RMB	0.99 (0.90,1.09)	1.02(0.93,1.12)	1.00 (0.91,1.10)	0.98 (0.89,1.09)	1.10 (0.96,1.27)
Current smoker	0.77 (0.72,0.83)	0.80 (0.74,0.86)	0.77 (0.71,0.82)	0.83 (0.77,0.90)	0.76 (0.68,0.86)
Current drinker	1.32 (1.23,1.41)	1.41 (1.31,1.51)	1.34 (1.25,1.43)	1.33 (1.23,1.43)	1.26 (1.13,1.41)
High fat diet	1.01 (0.95,1.07)	1.07 (1.01,1.13)	1.00 (0.94,1.06)	1.03 (0.96,1.10)	1.06 (0.97,1.16)
More vegetable and fruit intake	0.88 (0.84,0.92)	0.87 (0.83,0.91)	0.88 (0.84,0.92)	0.85 (0.81,0.89)	1.22 (1.14,1.31)
**Physical activity**					
Low	1.00	1.00	1.00	1.00	1.00
Moderate	0.74 (0.70,0.78)	0.73(0.69,0.77)	0.74 (0.70,0.78)	0.73 (0.69,0.77)	0.97 (0.89,1.05)
High	0.62 (0.59,0.66)	0.63 (0.60,0.67)	0.63 (0.59,0.66)	0.59 (0.56,0.63)	0.74 (0.68,0.81)

*Full model.

### Positively associated between MetS and cardiovascular disease

Table 3 showed associations between MetS and cardiovascular disease. Univariate analysis showed that MetS was positively associated with the stroke and CHD. After adjusted age, gender, culture, marriage status, income and current smoker, current drinker, high fat diet, vegetable, exercise, the results were not substantial changes.

**Table 3:** Association between MetS and cardiovascular disease (stroke and CHD).

	ATP β	IDF	JIS	CDS	EGIR
**Stroke**					
Model 1	2.17 (2.00,2.35)	1.64 (1.51,1.77)	2.14 (1.97,2.32)	1.78 (1.63,1.93)	1.90 (1.69,2.13)
Model 2	2.08 (1.91,2.26)	1.61 (1.47,1.75)	2.06 (1.89,2.24)	1.62 (1.48,1.77)	1.93 (1.72,2.17)
Model 3	2.00 (1.84,2.18)	1.56 (1.43,1.70)	1.99 (1.82,2.17)	1.56 (1.43,1.70)	1.87 (1.66,2.11)
**CHD**					
Model 1	1.76 (1.59,1.94)	1.56 (1.41,1.72)	1.78 (1.61,1.96)	1.59 (1.43,1.77)	1.92 (1.67,2.20)
Model 2	1.50 (1.35,1.66)	1.35 (1.22,1.50)	1.52 (1.37,1.68)	1.41 (1.27,1.57)	1.75 (1.52,2.00)
Model 3	1.49 (1.35,1.65)	1.36 (1.22,1.51)	1.51 (1.37,1.68)	1.42 (1.27,1.58)	1.70 (1.48,1.95)

Model 1: Non-adjusted;

Model 2: Adjusted for age, gender, culture, marriage status, income;

Model 3: Adjusted for model 2 plus current smoker, current drinker, high fat diet, vegetable, exercise.

## Discussion

The present survey were mainly focused on Chinese rural population which may provide important new evidence on the burden of MetS in China. The age-standardized prevalent MetS of the criteria of ATP β, IDF, JIS, CDS, and EGIR were 27.87%, 24.63%, 27.40%, 18.00%, and 8.91%, respectively. A total of 2106 participants were identified as MetS by the five criteria. There were negatively associations between ages, female, current drinker, and low physical activity with MetS.

In Beijing 2005, the prevalence of MetS was 28.5% (ATP β)^19^ which was similar to this study. Due to that the economic and urbanization development these years, the rural areas lifestyles become more likely the urban. In rural areas of Xinjiang province, the prevalence was 21.33% (IDF) in 2009^11^, which because of the multi-ethnic population distribution of the Uyghur, Hans, Kazakhs, and Kyrgyz, while in this study just for the Hans. According to the criteria of ATP β, IDF, CDS,the prevalence of Chinese adults was 21.3%, 18.2% and 10.5%^9^, which were lower than Henan province. The most reason was the study sample from nine provinces but in this study just from Henan province. However, Henan province was an agricultural province. In the criterion of CDS, the prevalence of MetS in male was higher than that in female, while the male was lower than female under other criteria, which was consistent with the study of Wanghong Xu^20^. The components of highest proportions were blood pressure and waist circumference, which was similar to the result of Jiangxi province of China^21^. Based on these analyses, in Henan rural areas, we could know that the prevalence of MetS maintained a high level, while the blood pressure and waist circumference would be the important aspects to prevent MetS.

The results showed that there was a negative relationship among ages, female, current drinker, no smoking, and low physical activity with MetS. About the factors, the most debatable factor was smoking. At the same time, one study had proven that smoking could lead to cardiovascular disorders^22^. This results might be due to the situations of individuals who had quit smoking and the influence of secondhand smoke. So that some people might quit smoking because of the health concern, which might result in a false appearance of smoking becoming a protective factor. However, there was an interesting phenomenon that more vegetable intake was a risk factor under the criterion of EGIR. As we all know, EGIR was a criterion that based on the insulin resistance, while the others criteria made the central obesity as a base. At the CDS and EGIR, the high level of culture was a protective factor, which was not found in other criteria. Addition, we observed that MetS was an independent risk factor for stroke and CHD. Evidence indicated that non-communicable disease (NCD) have been a leading cause of death, and about more than 40% NCD deaths were caused by CVD^23^. Several studies reported that MetS is a risk factor of stroke and CHD^24,25^. MetS play an important role in etiology of atherosclerosis and it is a key process of CVD^24,25^.

There were several limitations need to be mentioned. First, owing to nature of cross-sectional study, we could not demonstrate the causal relationships between factors and MetS. Therefore, future perspective studies are needed to confirm the results from this study. Second, according to the actual rural circumstances, the residents who studied or worked in city were not included in this study, so the prevalence of MetS might be overestimated in rural population. Third, although several important confound factors were controlled, there remain some other unselected or unmeasured covariates (such as genetic, air pollution and hydrocarbons factors) were not considered. Although these limitations existed in the study, the relatively large epidemiological study, to some extent, also could reflect the prevalence of MetS in rural areas of China.

## Conclusion

A higher prevalence of MetS in Chinese rural adults due to emerge from economic and social transition. More attention should be given to aged females with low physical activity and alcoholic consumption had a higher risk for MetS, which may be contributed to reduce the burden of MetS related stroke and coronary heart disease.

## Supplementary information

Supplementary File.

## Data Availability

All relevant data are within the paper and its Supporting Information files. Contact to Dr. Chongjian Wang (tjwcj2005@126.com) for additional information regarding data access.
