# Supplementary material for: Associations of unhealthy lifestyles with metabolic syndrome in Chinese rural aged females
Source: Sci Rep. 2020 Feb 17;10:2718. doi: 10.1038/s41598-020-59607-x (PMC7026414; doi:10.1038/s41598-020-59607-x)
Supplement: Supplementary file 1 — Supplementary File. [file 41598_2020_59607_MOESM1_ESM.pdf]

Title：

Associations of unhealthy lifestyles with metabolic syndrome in Chinese rural aged females

Authors:

Yuming Wang[a,b†], Runqi Tu[c†], Huijuan Yuan[a,b], Lijun Shen[a,b], Jian Hou[c], Xiaotian Liu[c], Miaomiao Niu[c], Zhihan Zhai[c], Mingming Pan[c], Chongjian Wang[c*]

Supplementary Table 1. The diagnosis criteria of MetS based on the different indexes.

criteria	ATPIII(2005)[4]	IDF(2005)[5]	JIS(2009)[6]	CDS(2013)[7]	EGIR(1999)[8]
Insulin resistance	None, but any 3 of the following 5 features	None	None, but any 3 of the following 5 features	None, but any 3 of the following 5 features	Plasma insulin>75th percentile plus any 2 of the following
Body weight	WC≥90cm in males;<br>≥80cm in females	Increased WC<br>(WC≥90cm in males or ≥80cm in females) plus any 2 of the following	WC≥90cm in males;<br>≥80cm in females	WC≥90cm in males;<br>≥80cm in females	WC≥94cm in males;<br>≥80cm in females
Triglycerides	≥1.7mmol/L<br>or on drug treatment for elevated triglycerides	≥1.7mmol/L<br>or specific treatment for this lipid abnormality	≥1.7mmol/L<br>or drug treatment for elevated triglycerides is an alternate indicator	≥1.7mmol/L	≥2.0mmol/L<br>or treated for dyslipidemia
HDL-C	<1.03mmol/L in males;<br><1.3mmol/L in females<br>or drug treatment for reduced HDL-C	<1.03mmol/Lin males;<br><1.29mmol/L in females<br>or specific treatment for this lipid abnormality	<1.03mmol/L in males;<br><1.29mmol/L in females<br>or drug treatment for reduced HDL-C is an alternate indicator	<1.04mmol/L	<1.0mmol/L<br>or treated for dyslipidemia
Blood pressure	≥130 mmHg systolic blood pressure or ≥85 mmHg diastolic blood pressure or on antihypertensive drug treatment in a patient with a history of hypertension	≥130 mmHg systolic blood pressure or ≥85 mmHg diastolic blood pressure or treatment of previously diagnosed hypertension	≥130 mmHg systolic blood pressure or ≥85 mmHg diastolic blood pressure or antihypertensive drug treatment in a patient with a history of hypertension is an alternate indicator	≥130 mmHg systolic blood pressure or ≥85 mmHg diastolic blood pressure or on antihypertensive drug treatment in a patient with a history of hypertension	≥140 mmHg systolic blood pressure or ≥90 mmHg diastolic blood pressure or treated for hypertension
Fasting glucose	≥100mg/dL<br>or on drug treatment for elevated glucose	≥5.6mmol/L<br>or previously diagnosed Type 2 diabetes	≥100mg/dL<br>or drug treatment of elevated glucose is an alternate indicator	≥6.1mmol/L<br>or previously diagnosed Type 2 diabetes	≥6.1 mmol/L, but nondiabetic

Abbreviations: WC, waist circumference; HDL-C, High density lipoprotein cholesterol;

Supplementary Table 2. Analysis on validity and consistencies of different criteria based on CDS for MetS.

indexes	ATPIII	JIS	IDF	EGIR
Sensitivity (%)	99.51	98.07	89.87	34.13
Specificity (%)	82.23	82.32	85.55	93.77
Youden's index	81.74	80.39	75.42	27.91
+LR	5.60	5.55	6.22	5.48
-LR	0.01	0.02	0.12	0.70
Kappa	0.68	0.68	0.67	0.33
Consistency rate (%)	86.31	86.04	86.57	82.39

Abbreviations: +LR, positive likelihood ratio; -LR, negative likelihood ratio.
